# The EZ diffusion model provides a powerful test of simple empirical effects

**DOI:** 10.3758/s13423-016-1081-y

**Published:** 2016-06-28

**Authors:** Don van Ravenzwaaij, Chris Donkin, Joachim Vandekerckhove

**Affiliations:** 1 University of Groningen, Department of Psychology, Grote Kruisstraat 2/1, Heymans Building, room 169, 9712 TS Groningen, The Netherlands; 2 University of New South Wales, Sydney, Australia; 3 University of California, Irvine, CA USA

**Keywords:** Sequential accumulator models, Response time analysis, Power, Model complexity

## Abstract

Over the last four decades, sequential accumulation models for choice response times have spread through cognitive psychology like wildfire. The most popular style of accumulator model is the diffusion model (Ratcliff *Psychological Review, 85*, 59–108, 1978), which has been shown to account for data from a wide range of paradigms, including perceptual discrimination, letter identification, lexical decision, recognition memory, and signal detection. Since its original inception, the model has become increasingly complex in order to account for subtle, but reliable, data patterns. The additional complexity of the diffusion model renders it a tool that is only for experts. In response, Wagenmakers et al. (*Psychonomic Bulletin & Review, 14*, 3–22, 2007) proposed that researchers could use a more basic version of the diffusion model, the EZ diffusion. Here, we simulate experimental effects on data generated from the full diffusion model and compare the power of the full diffusion model and EZ diffusion to detect those effects. We show that the EZ diffusion model, by virtue of its relative simplicity, will be sometimes better able to detect experimental effects than the data–generating full diffusion model.

**Electronic supplementary material:**

The online version of this article (doi:10.3758/s13423-016-1081-y) contains supplementary material, which is available to authorized users.

In everyday life, we are constantly confronted with situations that require a quick and accurate action or decision. Examples include mundane tasks such as doing the dishes (we do not want to break china, but we also do not want to spend the next hour scrubbing), or vacuum cleaning (we like to get as many nooks and corners as possible, but also want to get back to finishing that paper), but also more serious activities, such as typing a letter or performing a placement test. For all these actions, there exists a trade–off, such that greater speed comes at the expense of more errors. This phenomenon is called the speed–accuracy trade–off (Schouten and Bekker 1967; Wickelgren 1977).

In experimental psychology it is common practice to study this speed–accuracy trade–off with relatively simple tasks. More often than not, the task requires participants to decide between one of two alternatives as quickly and accurately as possible. Notable examples include the lexical decision paradigm (Rubenstein et al. 1970) in which the participant is asked to classify letter strings as English words (e.g., LEMON) or non–words (e.g., LOMNE), and the moving dots task (Ball and Sekuler 1982) in which participants have to determine whether a cloud of partly coherently moving dots appears to move to the left or to the right. Typically, the observed variables from these and other two–alternative forced choice tasks are distributions of response times (RTs) for correct and incorrect answers. One way to analyze the data from these kinds of tasks is to draw inferences based on one of, or both, the mean of the correct RTs, or the percentage of correct responses. These measures, however, do not speak directly to underlying psychological processes, such as the rate of information processing, response caution, and the time needed for stimulus encoding and non–decision processes (i.e., response execution). They also do not address the speed–accuracy trade–off.

The motivation among cognitive psychologists to be able to draw conclusions about these unobserved psychological processes has led to the advent of sequential accumulator models. A prominent example of such a model is the diffusion model (Ratcliff 1978). The model assumes that an observer accumulates evidence for responses until a threshold level of evidence for one of the responses is reached. The time taken to accumulate this evidence, plus a non–decision time, gives the observed response time, and the choice is governed by which particular threshold is reached.

Over the last four decades, as increasingly complex data patterns were observed, the diffusion model grew in order to account for these data. Ratcliff (1978) added the assumption that accumulation rate varied from trial to trial in order to account for the observation that incorrect responses were slower than correct responses. Ratcliff and Rouder (1998) assumed that the starting point of evidence could vary from trial to trial (following Laming, 1968), allowing them to account for incorrect responses that were faster than correct responses. Finally, Ratcliff and Tuerlinckx (2002) also proposed that non-decision time would vary across trials, an assumption that allowed the model to account for patterns in the speed with which the fastest responses were made.

The version of the diffusion model that includes all components of between–trial variability is known henceforth as the ‘full’ diffusion model. As a *theoretical* model of decision–making, the full diffusion model is impressive – it accounts for a wide range of reliable empirical phenomena. Among others, the diffusion model has been successfully applied to experiments on perceptual discrimination, letter identification, lexical decision, categorization, recognition memory, and signal detection (e.g., Ratclif,f 1978; Ratcliff et al., 2004, 2006; Klauer et al., 2007; Wagenmakers et al., 2008; van Ravenwaaij et al., 2011; Ratcliff et al., 2010). Using the diffusion model, researchers have examined the effects on decision making of alcohol intake (van Ravenzwaaij et al. 2012), playing video games (van Ravenzwaaij et al. 2014), sleep deprivation (Ratcliff and van Dongen 2009), anxiety (White et al. 2010), and hypoglycemia (Geddes et al. 2010). The model has also been applied extensively in the neurosciences (Ratcliff et al. 2007; Philiastides et al. 2006; Mulder et al. 2012).

In recent years, researchers have begun to use the full diffusion model as a *measurement* model. A diffusion model analysis takes as input the entire RT distribution for both correct and incorrect responses. The model maps the observed RTs and error rates into a space of psychological parameters, such as processing speed and response caution. Such an analysis has clear benefits over traditional analyses, which make no attempt to explain observed data in terms of psychologically meaningful processes.

A full diffusion model analysis is complicated, for two reasons. First, the model is complicated to use. Parameters for the model are estimated using optimization on functions that involve numerical integration and infinite sums.While there have been valiant efforts to make such models easier to use (Donkin et al., 2009, 2011; Vandekerckhove & Tuerlinckx, 2007, 2008; Voss & Voss, 2007), the application of a full diffusion model remains an approach most suited for experts. Second, the model itself may be more complex than is required by the data it is being used to fit, at least when the model is being used as a *measurement* model. When the data do not provide enough constraint on the estimation of model parameters, the more complex model will overfit the data, which leads to increased variability in parameter estimates.^1^

In response to the complexity of the full diffusion model, Wagenmakers et al. (2007) advocated for the use of the “EZ diffusion model”. The EZ diffusion model forgoes between–trial variability in accumulation rate, starting point, and non-decision time, as well as a–priori response bias (but see Grasman et al., 2009). By removing all of these additional model components, no fitting routine is required to estimate the parameters of the EZ diffusion model. Instead, the EZ diffusion takes RT mean, RT variance, and percentage correct, and transforms them into a mean rate of information accumulation, response caution, and a non–decision time.

The EZ model has been heralded for the ease with which it can be applied to data. However, critics have claimed that it is “too EZ” (Ratcliff, 2008, but see Wagenmakers et al., 2008). It is true that the EZ diffusion model can not account for the very broad range of data patterns for which the full diffusion was developed. However, the patterns of fast and slow errors, and shifting leading edges, that warrant the full complexity of the diffusion model are often observed in experiments that are specifically designed to observe such patterns, usually involving many thousands of trials. It is unclear whether such complex patterns can be detected in data coming from simpler experiments, at least to the point that they constrain the estimation of additional model parameters.

Van Ravenzwaaij and Oberauer (2009) examined the ability of both the full and EZ model to recover the mean structure and individual differences of parameter values used to generate fake data. The authors concluded that EZ was well capable of recovering individual differences in the parameter structure, but exhibited a bias in the recovery of the mean structure. Interestingly, the full diffusion model was unable to recover individual differences in the across–trial variability parameters, casting doubt on the added value of these extra parameters in more “typical” data sets. Recovery of the mean structure depended very much on the specific implementation.

Here, we show that the additional complexity of the full diffusion model has adverse consequences when one aims to use the model to *detect the existence of an empirical effect*. Simplifying the parametric assumptions of the diffusion model leads to increased precision in parameter estimation at the cost of possible bias due to model mis–specification (bias–variance trade–off; (Geman et al. 1992)). However, for the purposes of decision–making, bias is not necessarily detrimental (Gigerenzer and Brighton 2009) while higher precision leads to stronger and more accurate inference (Hastie et al. 2005). One of the aims of this manuscript is to help non–experts approach the notion of when to use the EZ model over the full diffusion model.

We simulate data in which we systematically vary the three main diffusion model parameters between two conditions: drift rate, boundary separation, and non-decision time. The data are simulated from a full diffusion model. We then show that, compared to the full diffusion model, the EZ diffusion model is the more powerful tool for identifying differences between two conditions on speed of information accumulation or response caution. We show that this holds across simulations that differ in the number of trials per participant, the number of participants per group, and the size of the effect between groups. We compare the proportion of times that the EZ and the full diffusion model detected a between–group effect on either mean speed of information accumulation, response caution, or non–decision time parameters (in terms of the result of an independent-samples *t*–test).

The remainder of this paper is organized as follows: in the next section, we discuss the diffusion model in detail. We examine the simple diffusion model, the full diffusion model, and EZ. In the section after that, we discuss our specific parameter settings for our simulation study. Then, we present the results of our simulations. We conclude the paper with a discussion of our findings and the implications for cognitive psychologists looking to analyze their data with the diffusion model.

## The diffusion model

**Fig. 1:**
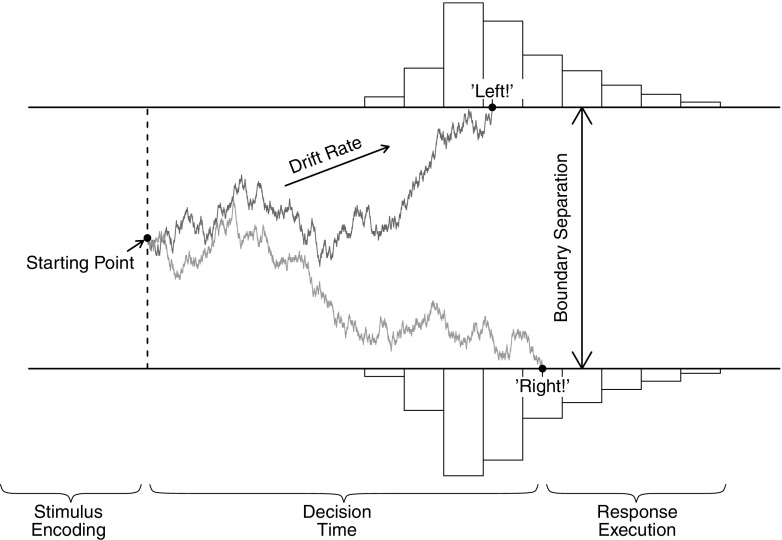
The diffusion model and its parameters as applied to a moving dots task (Ball and Sekuler 1982). Evidence accumulation begins at starting point *z*, proceeds over time guided by mean drift rate *ν*, and stops whenever the upper or the lower boundary is reached. Boundary separation *a* quantifies response caution. Observed RT is an additive combination of the time during which evidence is accumulated and non–decision time *T*_*er*_

In the diffusion model for speeded two–choice tasks (Ratcliff 1978; Vandekerckhove and Tuerlinckx 2007; van Ravenzwaaij et al. 2012), stimulus processing is conceptualized as the accumulation of noisy evidence over time. A response is initiated when the accumulated evidence reaches a predefined threshold (Fig. 1). The decision process begins at starting point *z*, after which information is accumulated with a signal–to–noise ratio that is governed by mean drift rate *ν* and within–trial standard deviation *s*.^2^ Mean drift rate *ν* values near zero produce long RTs and near–chance performance. Boundary separation *a* determines the speed–accuracy trade–off; lowering boundary separation *a* leads to faster RTs at the cost of more errors. Together, these parameters generate a distribution of decision times *DT*. The observed RT, however, also consists of stimulus–nonspecific components such as response preparation and motor execution, which together make up non–decision time *T*_*er*_. The model assumes that *T*_*er*_ simply shifts the distribution of *DT*, such that *R* *T* = *D* *T* + *T*_*er*_ (Luce 1986).

Hence, the four core components of the diffusion model are (1) the speed of information processing, quantified by mean drift rate *ν*; (2) response caution, quantified by boundary separation *a*; (3) a–priori response bias, quantified by starting point *z*; and (4) mean non–decision time, quantified by *T*_*er*_.

### Full diffusion

The simple diffusion model can account for most data patterns typically found in RT experiments, but has difficulty accounting for error response times that have a different mean from correct response times (Ratcliff 1978). One way for the model to produce slow errors is with the inclusion of across–trial variability in drift rate. Such variability will lead to high drifts that produce fast correct responses and low drifts that produce slow error responses.

One way for the model to produce fast errors is with the inclusion of across–trial variability in starting point (Laming 1968; Link 1975; Ratcliff and Rouder 1998). Such variability will cause most errors to happen because of an accumulator starting relatively close to the error boundary, whereas correct responses are still relatively likely to happen regardless of the starting point. As a consequence, the accumulated evidence will be lower on average for error responses than for correct responses, resulting in fast errors. For a more elaborate explanation of both of these phenomena, the reader is referred to Ratcliff and Rouder (1998, Fig. 2).

Thus, the full diffusion model includes parameters that specify across–trial variability in drift rate, *η*, and in starting point, *s*_*z*_. Furthermore, the model includes an across–trial variability parameter for non–decision time, *s*_*t*_, to better account for the leading edge of response time distributions (e.g., Ratcliff & Tuerlinckx, 2002).

### EZ diffusion

The EZ diffusion model presents the cognitive researcher with an alternative that does not require familiarity with complex fitting routines, nor does it require waiting a potentially long time on the model to estimate parameters from the data (Wagenmakers et al. 2007). All the researcher needs is to execute a few lines of code and the EZ parameters will be calculated instantaneously. The closed–form solutions for the EZ diffusion model require the assumption that there is no between–trial variability in drift rate, *η*, starting point, *s*_*z*_, or non–decision time, *s*_*t*_. Further, the model assumes that responses are unbiased (i.e., *z* is fixed at half of *a*).

The EZ diffusion model converts RT mean, RT variance, and percentage correct, into the three key diffusion model parameters: mean drift rate *ν*, boundary separation *a*, and non–decision time *T*_*er*_. The EZ diffusion model parameters are computed such that the error rate is described perfectly. EZ calculates diffusion model parameters for each participant and each condition separately. For applications of the EZ diffusion model, see e.g., Schmiedek et al. (2007), Schmiedek et al. (2009), Kamienkowski et al. (2011), and van Ravenzwaaij et al. (2012).

## Power simulations

We conducted four sets of simulations. For every set, we generated 4,500 data sets from the full diffusion model. All of the data sets were intended to mimic a two–condition between–subjects experiment. In the first three sets of simulations, we varied one of the three main diffusion model parameters systematically between the two groups. The fourth set of simulations was identical to the first, except we varied the mean starting point parameter.

The range of parameters we used were based on the distribution of observed diffusion model parameters, as reported in Matzke and Wagenmakers (2009). For Group 1, we sampled individual participant diffusion parameters from the following group distributions:

$$\begin{array}{@{}rcl@{}} \nu &\sim& N(0.223, 0.08)\mathrm{T}(0,0.586)\\ a &\sim& N(0.125, 0.025)\mathrm{T}(0.056,0.393)\\ T_{er}&\sim& N(0.435, 0.09)\mathrm{T}(0.206,0.942)\\ z &=& bias \times a\\ \eta &\sim& N(0.133, 0.06)\mathrm{T}(0.05,0.329)\\ s_{z} &\sim& N(0.3 \times a, 0.09 \times a)\mathrm{T}(0.05 \times a,0.9 \times a)\\ s_{t} &\sim& N(0.183, 0.037)\mathrm{T}(0,0.95 \times T_{er}) \end{array} $$

The notation ∼ *N*(,) indicates that values were drawn from a normal distribution with mean and standard deviation parameters given by the first and second number between parentheses, respectively. The notation T() indicates that the values sampled from the normal distribution were truncated between the first and second numbers in parentheses. Note that in the first three sets of simulations we fixed $bias=\frac {1}{2}$ in both the simulations and the model fits, reflecting an unbiased process such as might be expected if the different boundaries indicate correct vs. incorrect responding. In the fourth set of simulations, we relaxed this assumption and varied bias according to

$$\begin{array}{@{}rcl@{}} bias &\sim& N(0.5, 0.04)\mathrm{T}(0.25,0.75) \end{array} $$

For Group 2, all individual participant diffusion parameters were sampled from the same group distributions, except for either drift rate *ν* (sets 1 and 4), boundary separation *a* (set 2), or non-decision time *T*_*er*_ (set 3).^3^ For each parameter, we ran three different kinds of effect size simulations: a small, a medium, and a large effect size. Depending on the effect size, the Group 2 mean of the parameter of interest was larger than the Group 1 mean by 0.5, 0.8, or 1.2 within–group standard deviations for the small, the medium, and the large effect size, respectively. To illustrate for drift rate *ν*, depending on the simulation, we sampled individual participant diffusion parameters for Group 2 from the following group distributions:

$$\begin{array}{@{}rcl@{}} \nu_{small} &\sim& N(0.263, 0.08)\\ \nu_{medium} &\sim& N(0.287, 0.08)\\ \nu_{large} &\sim& N(0.319, 0.08) \end{array} $$

The small, medium, and large effect size mean parameters for boundary separation *a* and non-decision time *T*_*er*_ can be derived in a similar fashion. We varied the number of participants per group. The smallest group size was 10, the largest group size was 50, and we included all intermediate group sizes in steps of 10. We also varied the number of response time trials each participant completed: 50, 100, and 200.

Thus, to sum up, our simulations varied along the following dimensions:

1. Effect size: small (0.5 SD), medium (0.8 SD), and large (1.2 SD)
2. Number of participants: 10, 20, 30, 40, 50
3. Number of trials: 50, 100, 200

This resulted in a total of 45 types of simulations. We replicated each simulation type 100 times. We fit the resulting data with the full diffusion model, and we calculated EZ parameters. Next, we performed a *t*–test on the difference between the drift rate parameters in each of the two simulated groups, as estimated from the full diffusion and the EZ diffusion models. We recorded whether the obtained *p*–value was smaller than the traditional *α* of .05. Our analysis centers around the proportion of the 100 simulations for which the *p*-value was less than *α*.

### Results

**Fig. 2:**
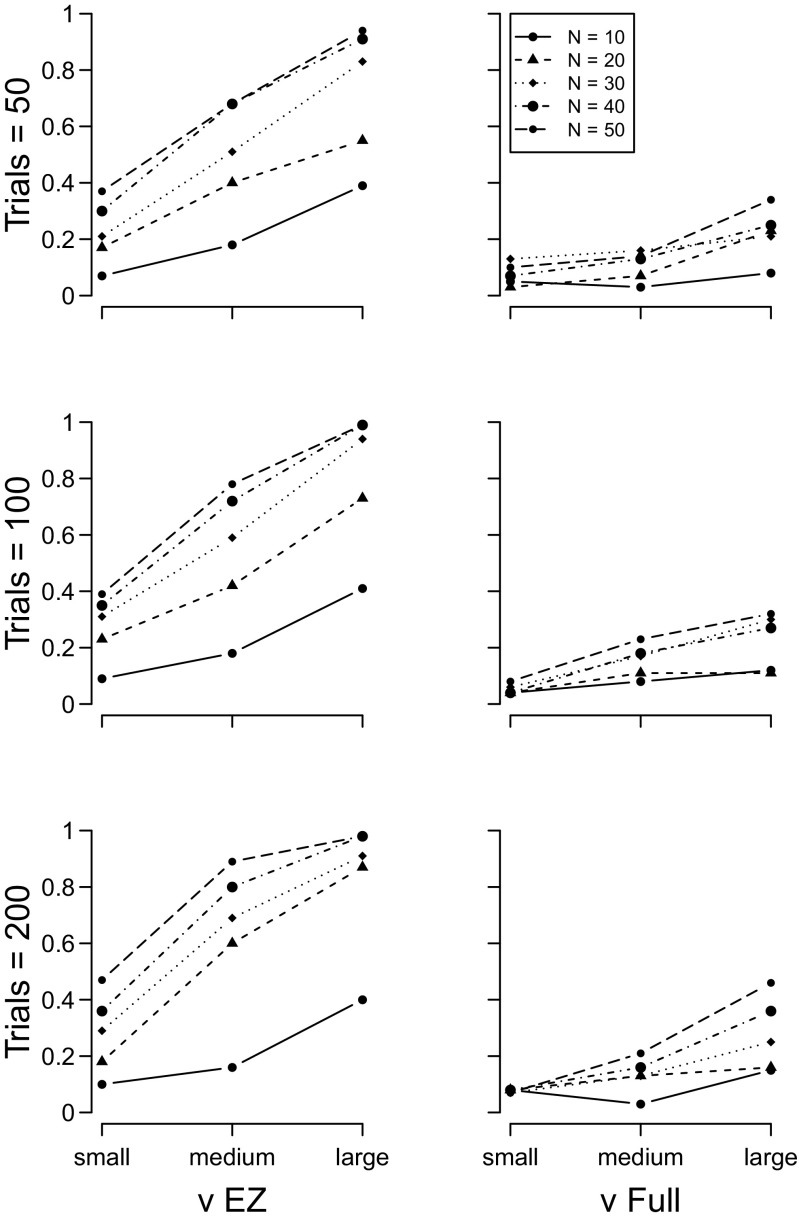
Proportion of times a significant between–group effect was detected with the diffusion model drift rate estimates. *Left column* = EZ diffusion *ν*; *right column* = full diffusion *ν*. *Top row* = 50 trials, *middle row* = 100 trials, *bottom row* = 200 trials. *Different lines* indicate different numbers of participants per group

**Fig. 3:**
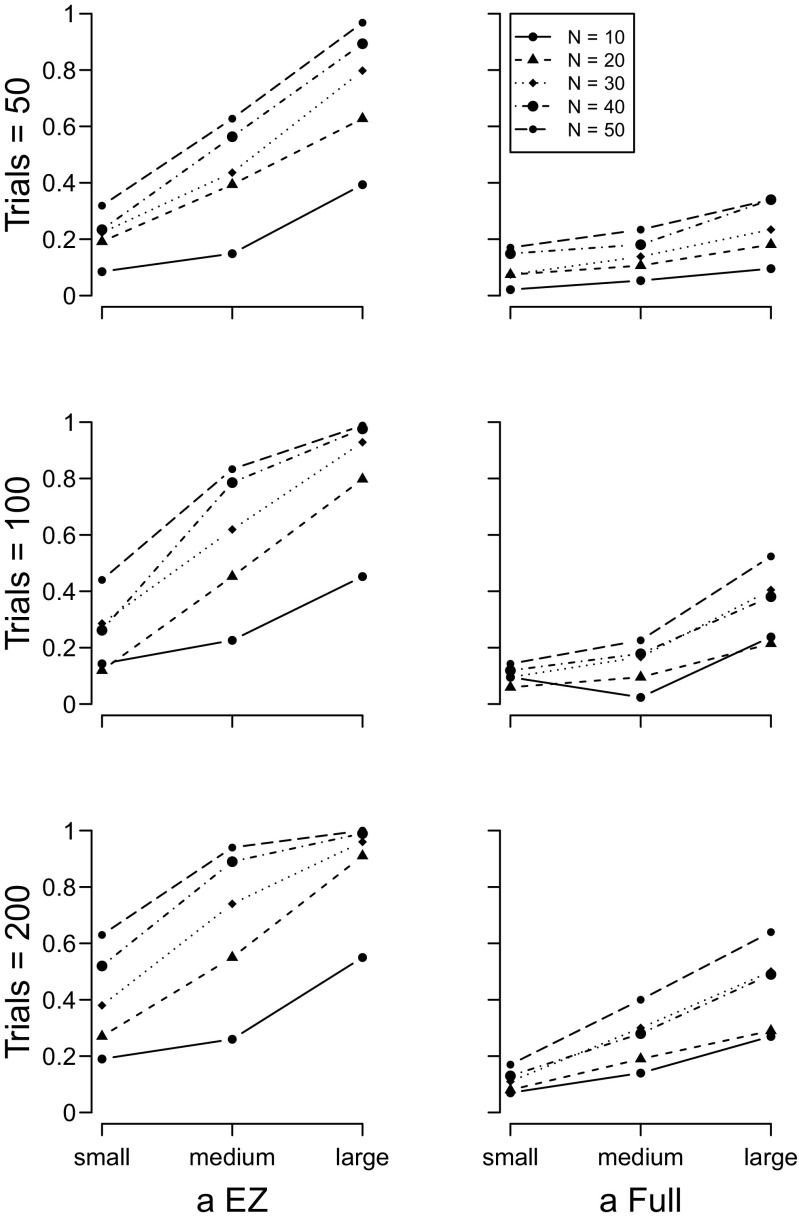
Proportion of times a significant between–group effect was detected with the diffusion model boundary separation estimates. *Left column* = EZ diffusion *a*; *right column* = full diffusion *a*. *Top row* = 50 trials, *middle row* = 100 trials, *bottom row* = 200 trials. *Different lines* indicate different numbers of participants per group

**Fig. 4:**
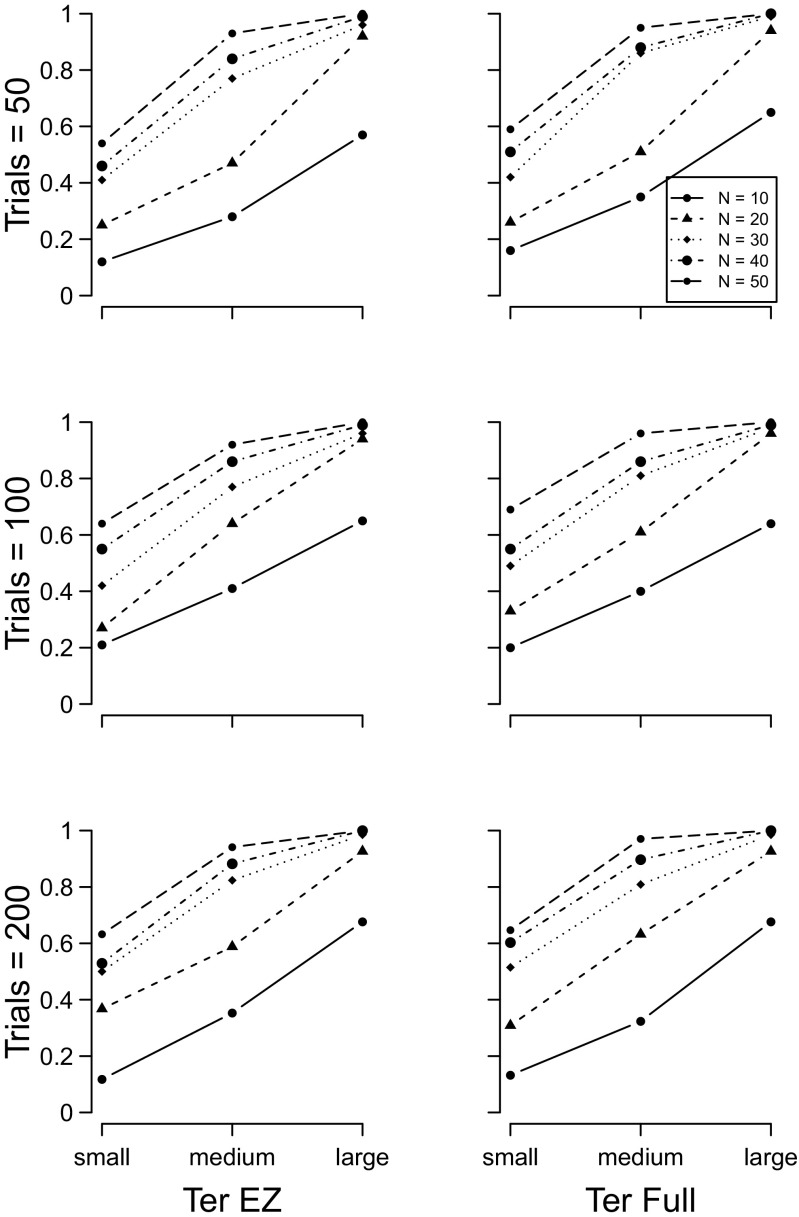
Proportion of times a significant between–group effect was detected with the diffusion model non–decision time estimates. *Left column* = EZ diffusion *T*_*er*_; *right column* = full diffusion *T*_*er*_. *Top row* = 50 trials, *middle row* = 100 trials, *bottom row* = 200 trials. *Different lines* indicate different numbers of participants per group

The results of the drift rate *ν*, boundary separation *a*, and non-decision time *T*_*er*_ simulations (sets 1 to 3) are shown in Figs. 2, 3, and 4, respectively. In all plots, the y–axis plots the proportion of 100 simulations for which a *t*–test on the focal parameter of the two groups yielded a *p*<.05. The results for the EZ diffusion model are plotted in the left column, and the full diffusion in the right column. For both models, the number of participants in each group increases the power of the analysis, as does the number of trials per participant.

For both drift rates and boundary separation parameters, the EZ diffusion model provides a higher-powered test than does the full diffusion model. When the two groups differed in terms of non-decision time, the EZ and full diffusion models perform equivalently.

**Fig. 5:**
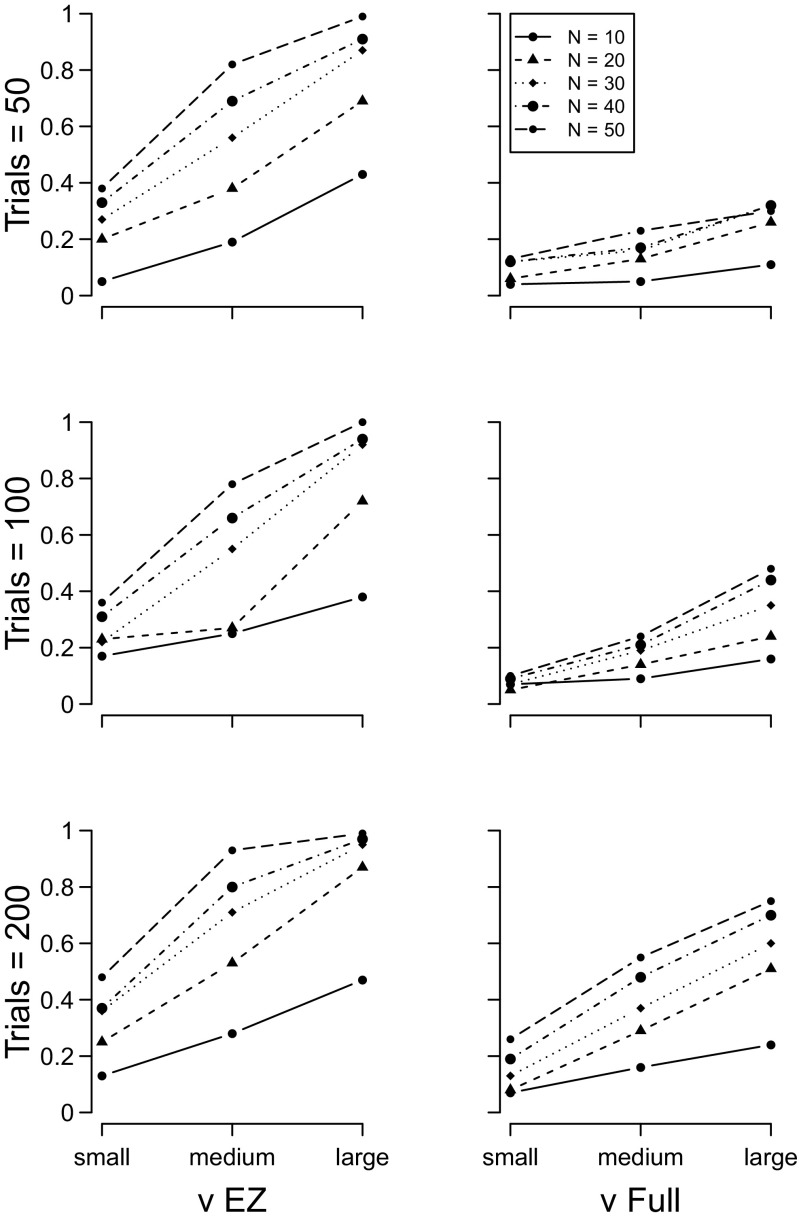
Proportion of times a significant between–group effect was detected with the diffusion model drift rate estimates. *Left column* = EZ diffusion *ν*; *right column* = full diffusion *ν*. *Top row* = 50 trials, *middle row* = 100 trials, *bottom row* = 200 trials. *Different lines* indicate different numbers of participants per group

Finally, the results of the drift rate with start point bias simulations (set 4) are shown in Fig. 5. We see that the results of the set 1 simulations are mirrored for set 4. Though the full diffusion model now fares slightly better than before in terms of power, the EZ diffusion model still provides a more powerful test of the difference between drift rates. That is, even when starting point bias is allowed to vary, the EZ diffusion model detects a difference between the two groups more often than does the full diffusion model.

Perhaps the higher power of the EZ diffusion model comes at the expense of a higher Type 1 error rate for the other parameters? In order to investigate this potential caveat, we have done the same kind of analyses for the two non–focal diffusion model parameters. That is, for sets 1 and 4, we looked at the proportion of times an effect was found for boundary separation and non–decision time and compared these results for the EZ model and the full diffusion model. For set 2, we made this comparison for drift rate and non–decision time. For set 3, we compared drift rate and boundary separation. Detailed results can be found in the Supplementary Material, available on www.donvanravenzwaaij.com/Papers. The conclusion is that the type 1 error rate is very low and comparable for both models for all simulation sets.

When taken together, the difference between the two models is striking. This result is probably quite surprising, given that the data were generated using the full diffusion model. The full diffusion model should have an advantage, but the complexity of the model turns out to impair its ability to detect effects, even when compared to models that are simplifications of the generating model.

## Discussion

The result of our simulations is simple: EZ diffusion is a more powerful tool than the full diffusion model when attempting to detect a between–group effect on speed of information processing or response caution. One potential explanation for this result is that the parameters of the full diffusion model are not well–constrained by the data from a single condition. We simulated data from a model in which only drift rate differed, on average, between the two groups. However, when the full diffusion model was fit to the data from an individual, then its six free parameters (*ν*, *a*, *T*_*er*_, *η*, *s*_*z*_, *s*_*t*_) varied in such a way to ‘overfit’ the data. This additional variability in parameter estimates led to a reduction in the power of the test comparing just the *ν*, *a* or *T*_*er*_ parameters of the two groups.

We now discuss a number of possible alternative explanations and caveats for our results.

### Optimization versus calculation

A difference between the two approaches is that we use a fitting routine to obtain the parameters of the full diffusion model, while the EZ diffusion model utilizes closed–form estimates of the model parameters. Here, we used the fastDM (Voss and Voss 2008) code in conjunction with Quantile Maximum Proportion Estimation (Heathcote et al. 2002) to fit the full diffusion model. The starting points of the optimization algorithm were the true population–level mean values, and SIMPLEX was used with a total of 2,500 steps. It is possible that the results we obtained were caused because we were unable to find the best set of parameter values with the full diffusion model.

To determine the extent to which parameter estimation was an issue, we used the same method to estimate the parameters of the EZ diffusion model, instead of relying on the closed–form solutions. Put differently: we estimated parameters for the simple diffusion model and compared its power to that of EZ and full diffusion for the drift rate simulations. The result is almost identical power for the EZ and simple diffusion models.^4^

The problem of optimization is less pronounced for the simple diffusion model than for the full diffusion model, because it has fewer parameters. That is, the optimizer will more often find the best–fitting parameter estimates because there are fewer parameters to optimize. However, the fact that the results of EZ and simple diffusion are so similar makes it, in our opinion, quite unlikely for this pattern to emerge entirely as a result of optimization issues. On top of that, even if the result were caused by poorer estimation of the full diffusion model, it still might be preferable to subvert this problem entirely, and simply use the EZ diffusion model.

It is also important to stress again here that for our simulations, the data–generating process was the full diffusion model. When applying the models to real data, both models become misspecified. As such, even perfect optimization would not necessarily lead to higher power for the full diffusion model.

### Parameter estimates will be biased

One issue with using the EZ diffusion model is that the parameter estimates of the model are systematically biased when the full diffusion model is used as the data generating process. As such, if the full diffusion model does provide an accurate representation of the way in which decisions are made, then one should interpret the actual values estimated by the EZ diffusion model with care. In other words, it is important to clarify that our positive assessment of the EZ diffusion model is with respect to its statistical power, and not with its estimation properties (see above comments about the bias-variance trade-off). If the aim of one’s research project is an unbiased estimate of full diffusion model parameters, then one needs to conduct a very different kind of experiment to the one simulated here (more on that in the next section). However, the model comparison approach is especially appropriate if the primary interest is in the discovery of general laws and invariances (e.g., Rouder et al., 2015).

Of course, issues with EZ’s unbiased estimates of the full diffusion model should be taken with a grain of salt, since it seems exceedingly unlikely that the full diffusion *is* the data–generating process. As such, the real question is whether the degree to which the bias in the parameter estimates of the full diffusion model is smaller than that of the EZ diffusion model, with respect to the true data–generating process. Almost half a decade of research tells us that the full diffusion model is a better representation of the decision–making process than the EZ diffusion, but it seems unlikely that the full diffusion model is actually the true data–generating process. For example, response thresholds appear to sometimes decrease over time (Hawkins et al. 2015; Zhang et al. 2014), evidence appears to leak with time (Usher and McClelland 2001), and the drift rate is not always a stationary signal (Smith and Ratcliff 2009). It is unclear to what extent the full diffusion parameter estimates are biased because the model does not incorporate these factors, not to mention the factors not yet identified.

### More complex designs

The design of our simulation study was remarkably simple. Our simulations were limited to two between–subjects conditions. It is lore among the choice response time model community that such a design is unlikely to yield reliable parameter estimates. As such, to those readers, our results are presumably not overly surprising. Our message, and therefore the series of simulations, is not meant for an expert audience. Our design was meant to reflect the kind of experiment that was not necessarily developed with response time models in mind.

The diffusion model is becoming increasingly often used as a post–hoc *measurement* model. A simple, between–subjects analysis represents the kind of study to which these models are being applied. Our message is rather that if it is not the researcher’s goal to explain the detailed shape of their response time distributions but rather to infer simple differences between conditions, then they are probably better served with an EZ diffusion model analysis, rather than one in which all parameters of the full diffusion model are estimated separately across conditions.

To get the best results of a choice response time model analysis, however, researchers should consult existing tutorials before running their studies (e.g., Voss et al., 2015). The advice will be to have experimental conditions across which parameters are not expected to vary. By constraining some of the parameters of the model across experiment conditions, it becomes possible to constrain even the between–trial variability parameters of the full diffusion model. We speculate that if we were to repeat our simulations with multiple experimental conditions, and constrain most of the parameters of the full diffusion model across those conditions, that the power of the full diffusion model analysis would increase.

### Hierarchical Bayesian methods

We have taken a frequentist approach in this manuscript – obtaining best–fitting parameters, and subjecting them to null–hypothesis significance tests. This choice was made in order to best mimic the approach likely to be taken by someone new to choice response time models. We prefer an alternative approach. First, we prefer to use hierarchical models (e.g., Vandekerckhove et al., 2011), in which parameters are estimated at the population–level, as informed by individuals that are assumed to conform to a particular statistical distribution (e.g., individual participant drift rates are normally distributed). Second, rather than obtaining a single set of best–fitting parameters, we prefer to think about posterior distributions, which allow for uncertainty in parameter values. Finally, we would prefer to use Bayes factors to compare model parameters. For example, for the design we used here, one could obtain posterior distributions for the population–level mean drift rates for each group, and then perform a Savage–Dickey test on the difference between those two drift rates (Wagenmakers et al. 2010).

For those willing to explore (slightly) more complicated methods, a hierarchical Bayesian approach is worth the effort. However, our general point that simpler models provide more precise parameter estimates carries the same implications for Bayesian analyses. For example, if one were to calculate Bayes factors based on the *t*–statistics we used in our hypothesis tests, then a similar conclusion would be reached. Further, models with fewer unnecessary parameters will also yield narrower posterior distributions, and so yield more conclusive Savage–Dickey Bayes factors.

## Conclusion

What are we to learn from this? If researchers are interested in maximizing the power of their design, analyzing their data with the full diffusion model is not always the best approach. If the full diffusion model does not provide the highest power even when data are generated by the full diffusion model, it is unlikely that the full diffusion model would do much better with real data. These results complement the results of van Ravenzwaaij and Oberauer (2009), who found that the full diffusion model was unable to recover individual differences in the across–trial variability parameters. The full diffusion model provides a powerful description of the full range of processes underlying performance in speeded decision making tasks. Perhaps, we presently lack the tools to collect data rich enough for the specialized full diffusion model to outshine his more parsimonious competitor.

We demonstrated that the cognitive researcher who is interested in a powerful design for detecting experimental effects in their RT tasks should analyze their data with relatively simple versions of the diffusion model. Even in the land of RT research, sometimes less is more.

## Electronic supplementary material

Below is the link to the electronic supplementary material.

(ZIP 205 KB)

## Footnotes

1 To see this, imagine an experimental design in which the between-trial variability in accumulation rate parameter in the diffusion model, *ν*, is unidentifiable (i.e., every value of $\hat {\nu }$ can yield the same likelihood value). If we were to fit a model to data that includes *ν*, the maximum likelihood value of the other parameters in the model, *𝜃*, will be estimated conditional on $\hat {\nu }$. Because the parameters in the diffusion model are correlated, the value of $\hat {\theta }$ depends on $\hat {\nu }$. As such, estimating *ν* artificially increases the variability in estimates of *𝜃*.

2 Mathematically, the change in evidence *X* is described by a stochastic differential equation *d* *X*(*t*) = *ν*⋅*d* *t* + *s*⋅*d* *W*(*t*), where *s*⋅*d* *W*(*t*) represents the Wiener noise process with mean 0 and variance *s* ^2^⋅*d* *t*. The standard deviation parameter *s* is often called the “diffusion coefficient” and serves as a scaling parameter that is often set to 0.1 or to 1.

3 We chose not to include a simulation in which the bias parameter was the only parameter that varied systematically between conditions. We expect that the EZ model will incorrectly attribute the difference in data to one of the three other parameters, and therefore lead to an incorrect conclusion. Our recommendation is for the researcher who expects response bias to vary across conditions to use the full diffusion model, or the EZ2 model (Grasman et al. 2009).

4 A figure with the results of this simulation is available in the Supplementary Material that can be obtained from www.donvanravenzwaaij.com/Papers.
