# Supplementary material for: The EZ diffusion model provides a powerful test of simple empirical effects
Source: Psychon Bull Rev. 2016 Jun 28;24(2):547–56. doi: 10.3758/s13423-016-1081-y (PMC5389995; doi:10.3758/s13423-016-1081-y)
Supplement: Supplementary file 1 — (ZIP 205 KB) [file 13423_2016_1081_MOESM1_ESM.zip › PowerSup.pdf]

SUPPLEMENTARY MATERIAL

The EZ Diffusion Model Provides a Powerful Test of Simple Empirical Effects

Don van Ravenzwaaij[1], Chris Donkin[2], and Joachim Vandekerckhove[3]
[1]University of Groningen, [2]University of New South Wales, [3]University of California, Irvine

Correspondence concerning this article should be addressed to:
Don van Ravenzwaaij
University of Groningen, Department of Psychology
Grote Kruisstraat 2/1, Heymans Building, room 169
9712 TS Groningen, The Netherlands
Ph: (+31) 50 363 7021
E–mail should be sent to d.van.ravenzwaaij@rug.nl.

This document consists of two sections. The first section contains the results of the type 1 error analyses of the four sets of simulations presented in van Ravenzwaaij, Donkin, and Vandekerckhove (2016). The second section compares performance of the EZ model to performance of the simple diffusion model for the first set of simulations.

Type 1 Error Analyses

Details of the four power simulations can be found in van Ravenzwaaij et al. (2016). The paper contains power analyses for the focal parameters (drift rate, boundary separation, non–decision time, and drift rate again, respectively). Here, we present results for the non–focal parameters.

The results of simulation sets 1 to 4 are shown in Figures 1, 2, 3, and 4, respectively. In all plots, the y–axis plots the proportion of 100 simulations for which a t–test on the non–focal parameter of the two groups yielded a $p < .05$. The results for the EZ diffusion model are plotted in the first and third column, and the full diffusion in the second and fourth column. In general, for both models the type 1 error rate is low. EZ error rates for drift rate ν and, to a lesser extent, non–decision time T_{er} are slightly elevated for simulation set 2 for large effect sizes on the boundary separation a parameter (Figure 2). In the most extreme cases, the EZ error rates are about 0.1 higher than the full model error rates. Note that these are the scenarios for which the EZ diffusion model has the most pronounced advantage over the full diffusion model in terms of power where the focal parameter is concerned (see Figure 3 of van Ravenzwaaij et al., 2016).

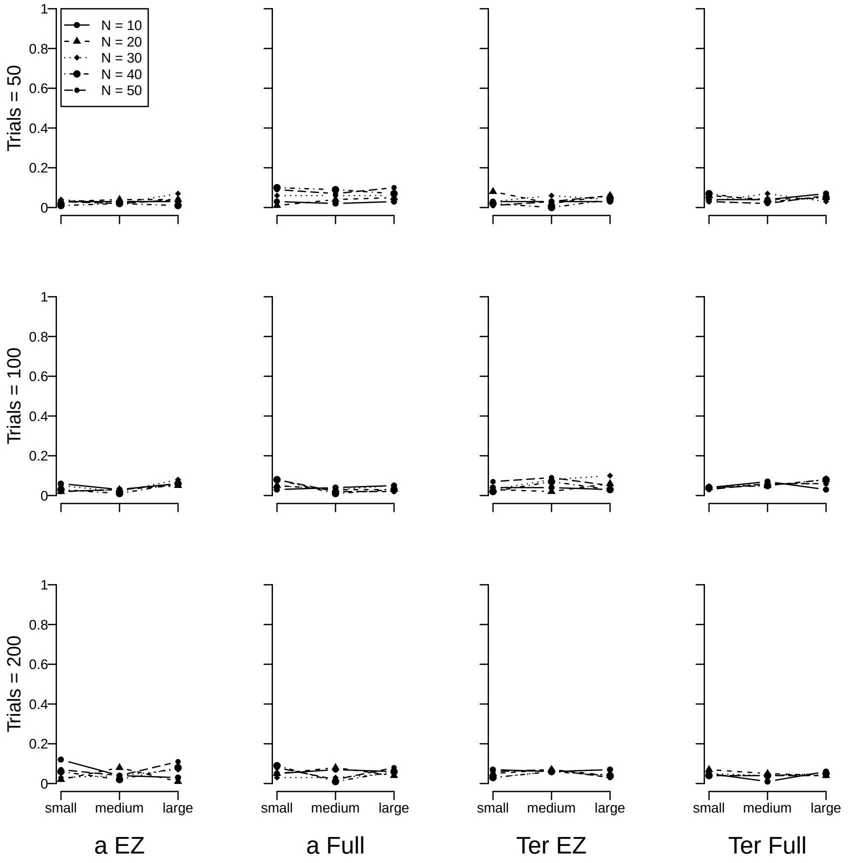

Figure 1. Proportion of times a significant between–group effect was detected for a non–focal parameter in simulation set 1. First and third column = EZ diffusion; second and fourth column = full diffusion; left two columns = boundary separation a; right two columns = non–decision time T_{er}. Top row = 50 trials, middle row = 100 trials, bottom row = 200 trials. Different lines indicate different numbers of participants per group.

EZ Diffusion Versus Simple Diffusion

The power results of the drift rate ν simulation (set 1) with the simple diffusion model included are shown in Figure 5. The results show that EZ and the simple diffusion model perform comparable, both outperform the full diffusion model.

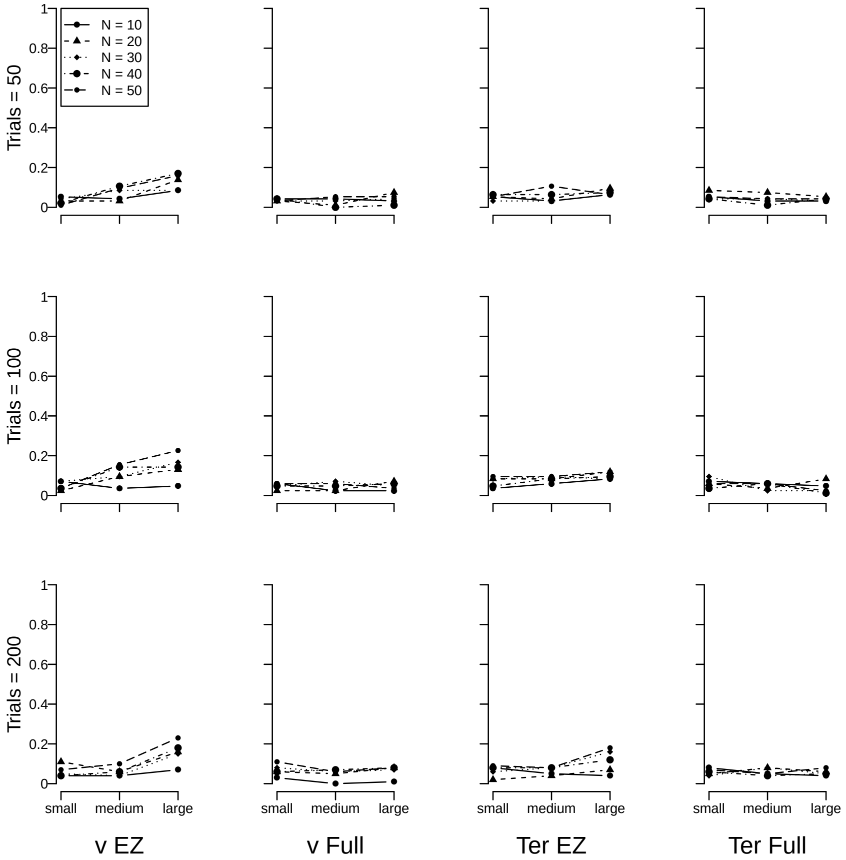

Figure 2. Proportion of times a significant between–group effect was detected for a non–focal parameter in simulation set 2. First and third column = EZ diffusion; second and fourth column = full diffusion; left two columns = drift rate ν; right two columns = non–decision time T_{er}. Top row = 50 trials, middle row = 100 trials, bottom row = 200 trials. Different lines indicate different numbers of participants per group.

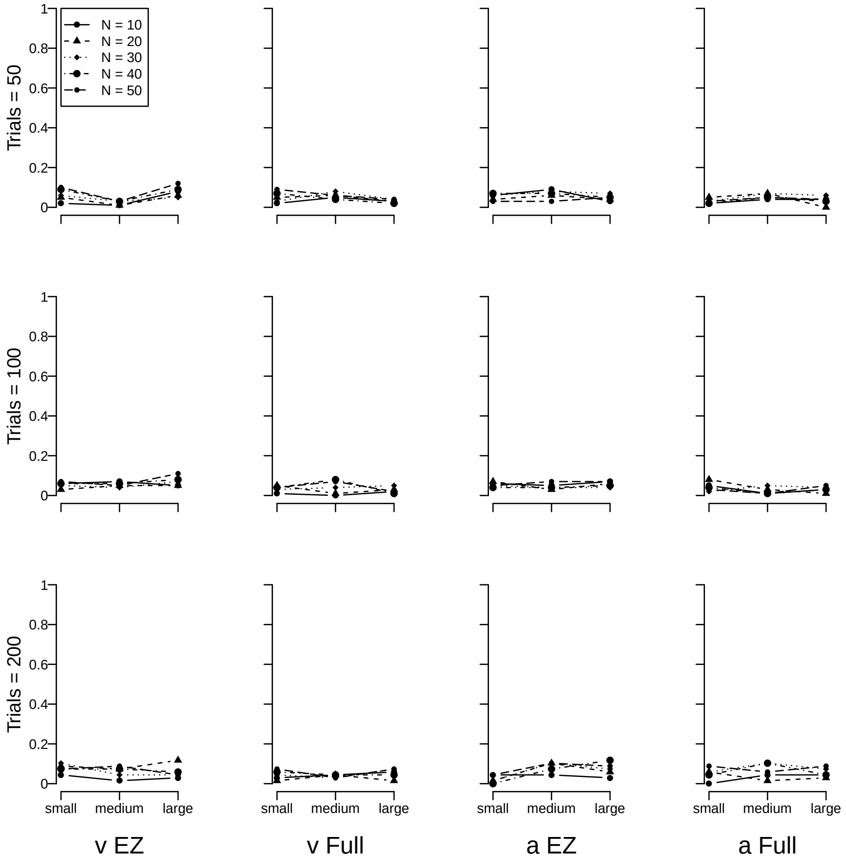

Figure 3. Proportion of times a significant between–group effect was detected for a non–focal parameter in simulation set 3. First and third column = EZ diffusion; second and fourth column = full diffusion; left two columns = drift rate ν; right two columns = boundary separation a. Top row = 50 trials, middle row = 100 trials, bottom row = 200 trials. Different lines indicate different numbers of participants per group.

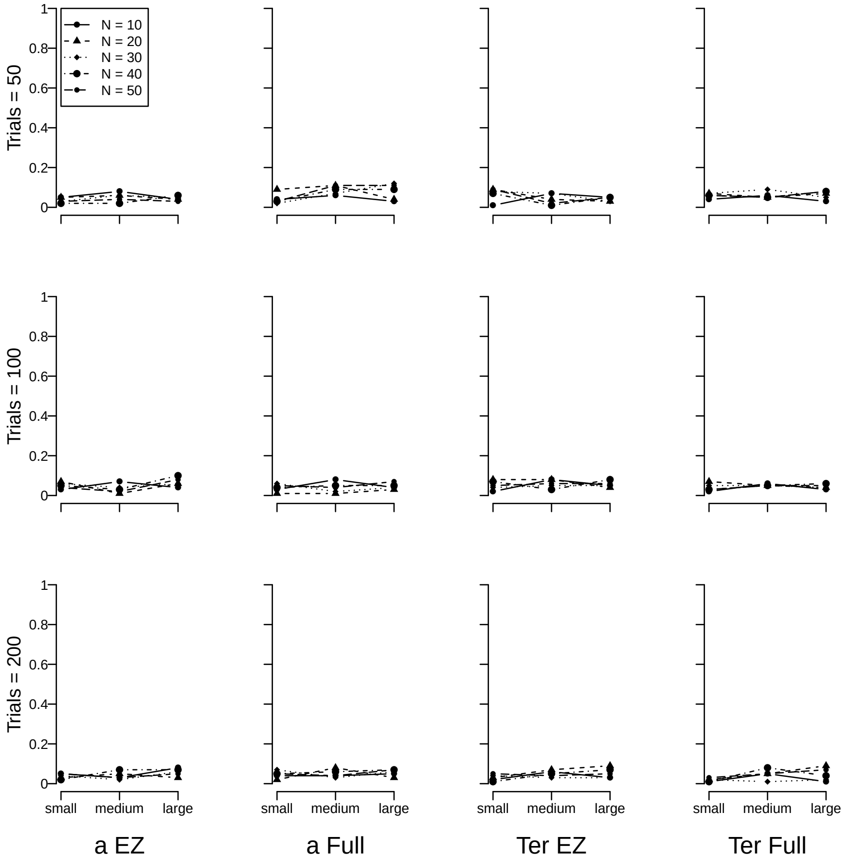

Figure 4. Proportion of times a significant between–group effect was detected for a non–focal parameter in simulation set 4. First and third column = EZ diffusion; second and fourth column = full diffusion; left two columns = boundary separation a; right two columns = non–decision time T_{er}. Top row = 50 trials, middle row = 100 trials, bottom row = 200 trials. Different lines indicate different numbers of participants per group.

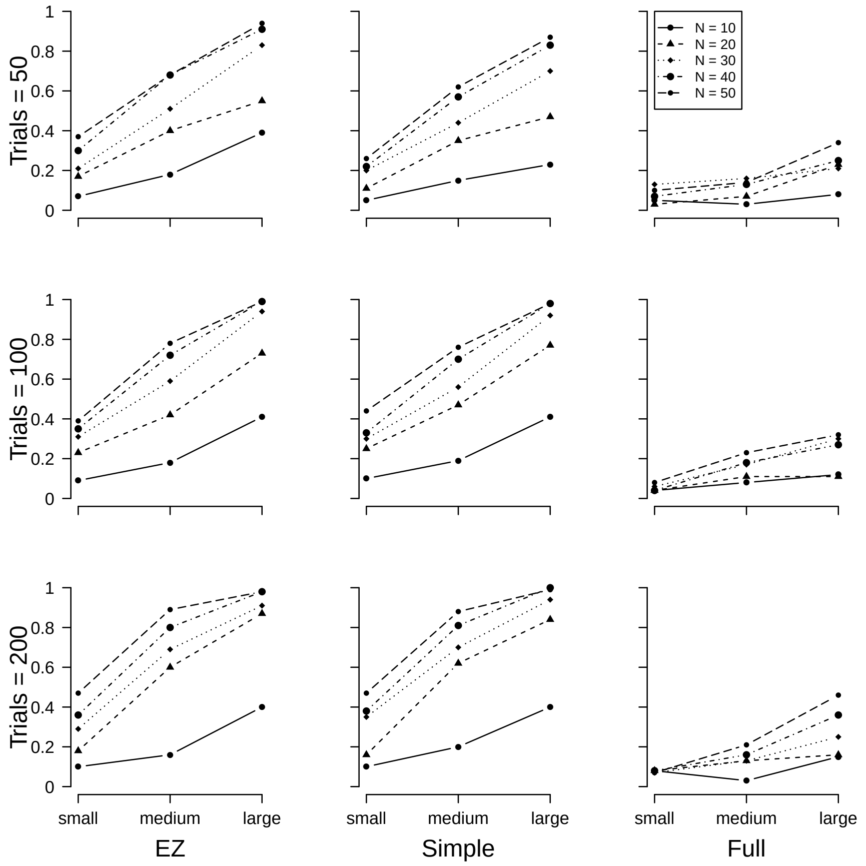

Figure 5. Proportion of times a significant between–group effect was detected with the diffusion model drift rate estimates. Left column = EZ diffusion ν; middle column = simple diffusion ν; right column = full diffusion ν. Top row = 50 trials, middle row = 100 trials, bottom row = 200 trials. Different lines indicate different numbers of participants per group.

References

van Ravenzwaaij, D., Donkin, C., & Vandekerckhove, J. (2016). The EZ Diffusion model provides a powerful test of simple empirical effects. Manuscript submitted for publication.
