# Supplementary figures and images for: The EZ diffusion model provides a powerful test of simple empirical effects
Source: Psychon Bull Rev. 2016 Jun 28;24(2):547–56. doi: 10.3758/s13423-016-1081-y (PMC5389995; doi:10.3758/s13423-016-1081-y)

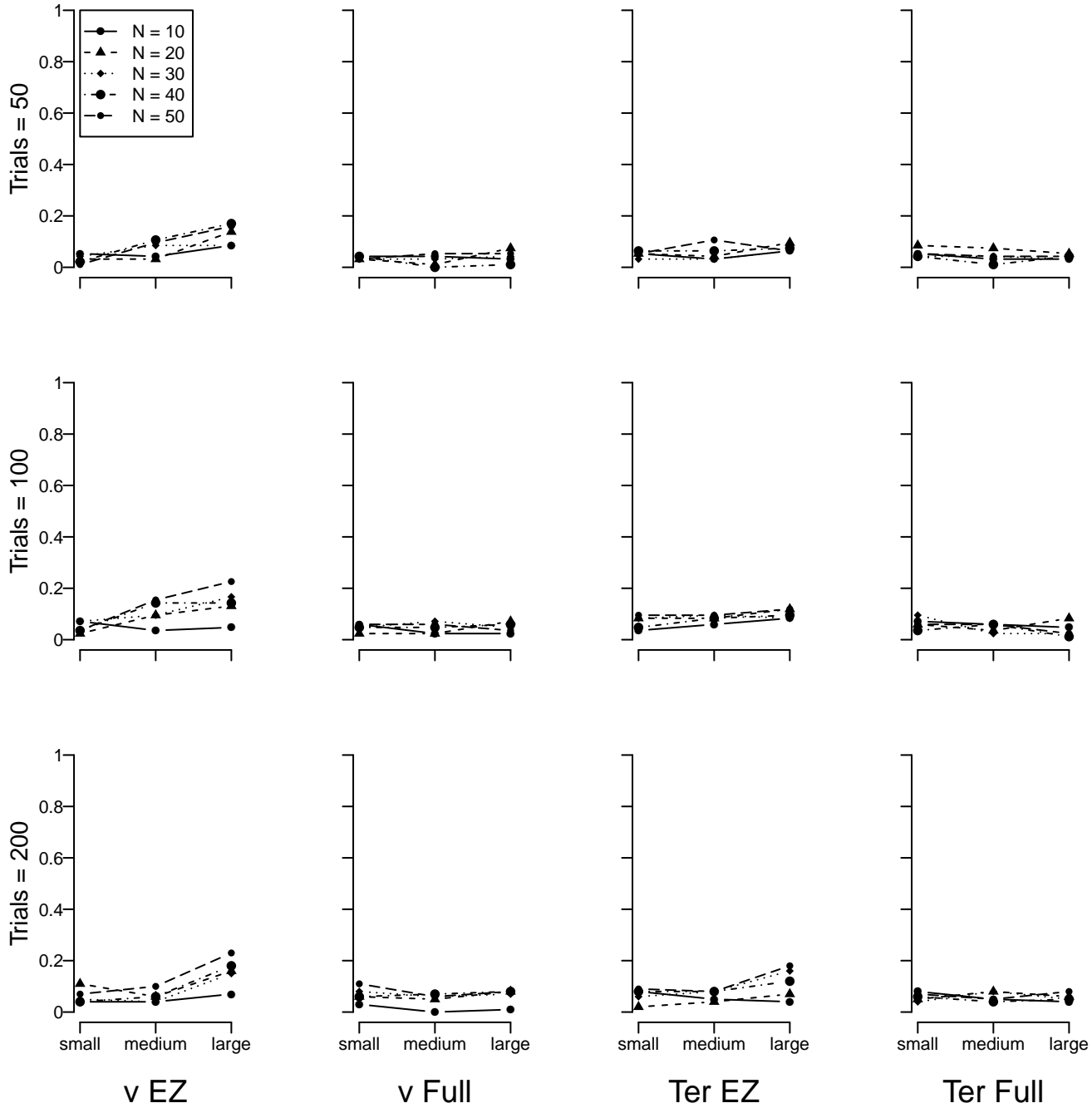

Supplement: Supplementary file 1 — (ZIP 205 KB) [file 13423_2016_1081_MOESM1_ESM.zip › Figures/aFullRes.pdf]

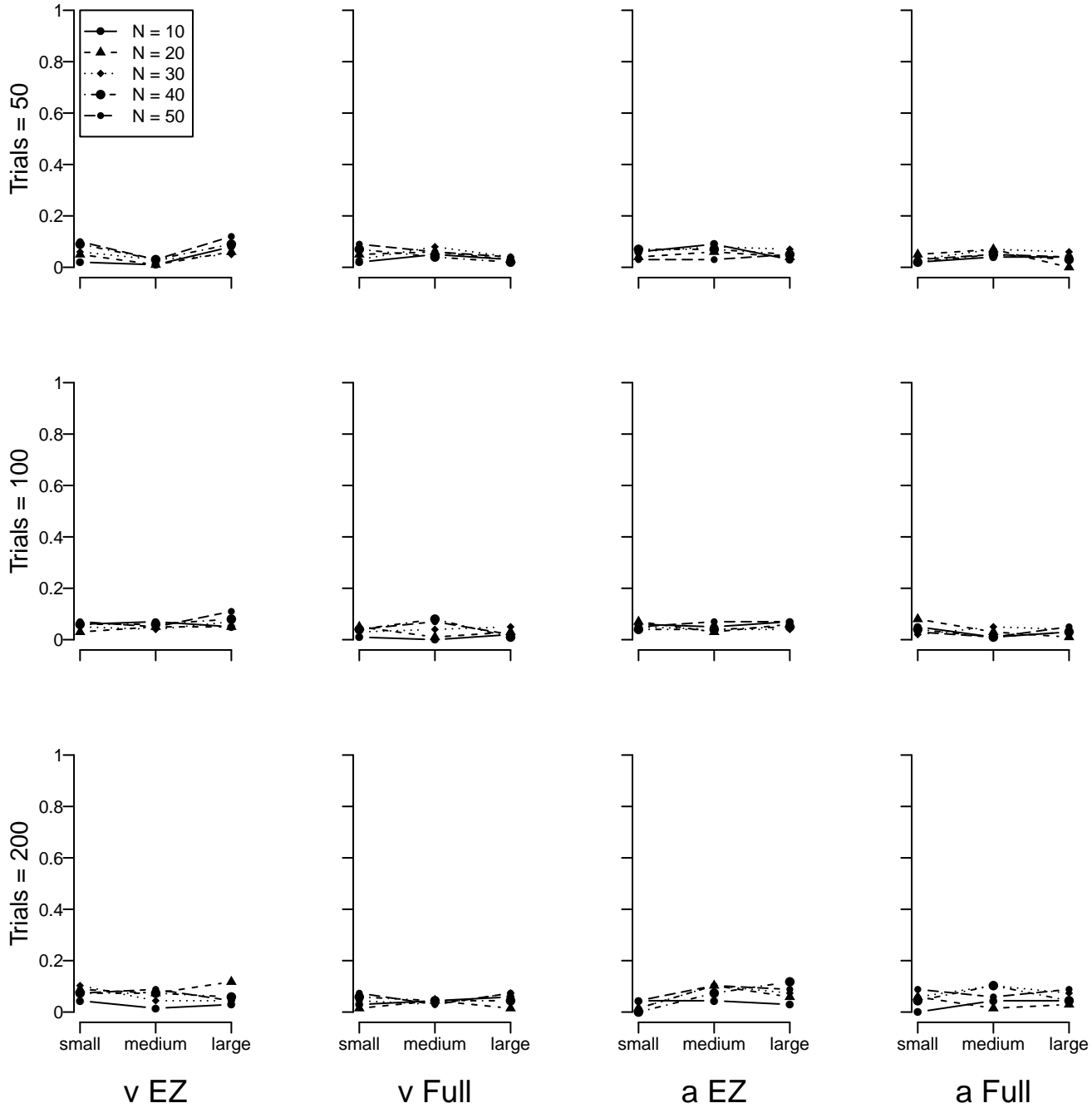

Supplement: Supplementary file 1 — (ZIP 205 KB) [file 13423_2016_1081_MOESM1_ESM.zip › Figures/terFullRes.pdf]

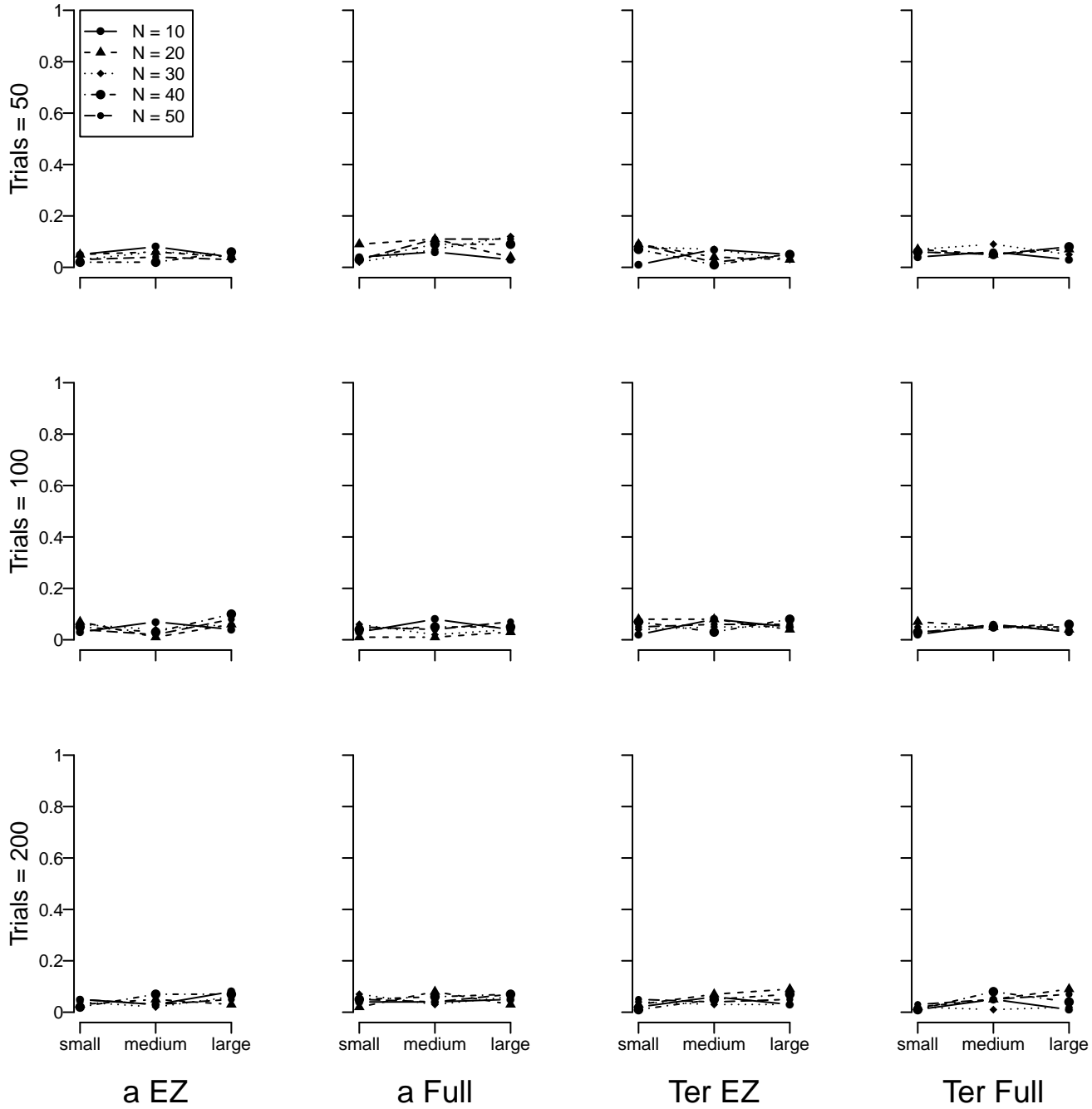

Supplement: Supplementary file 1 — (ZIP 205 KB) [file 13423_2016_1081_MOESM1_ESM.zip › Figures/vBiasFullRes.pdf]

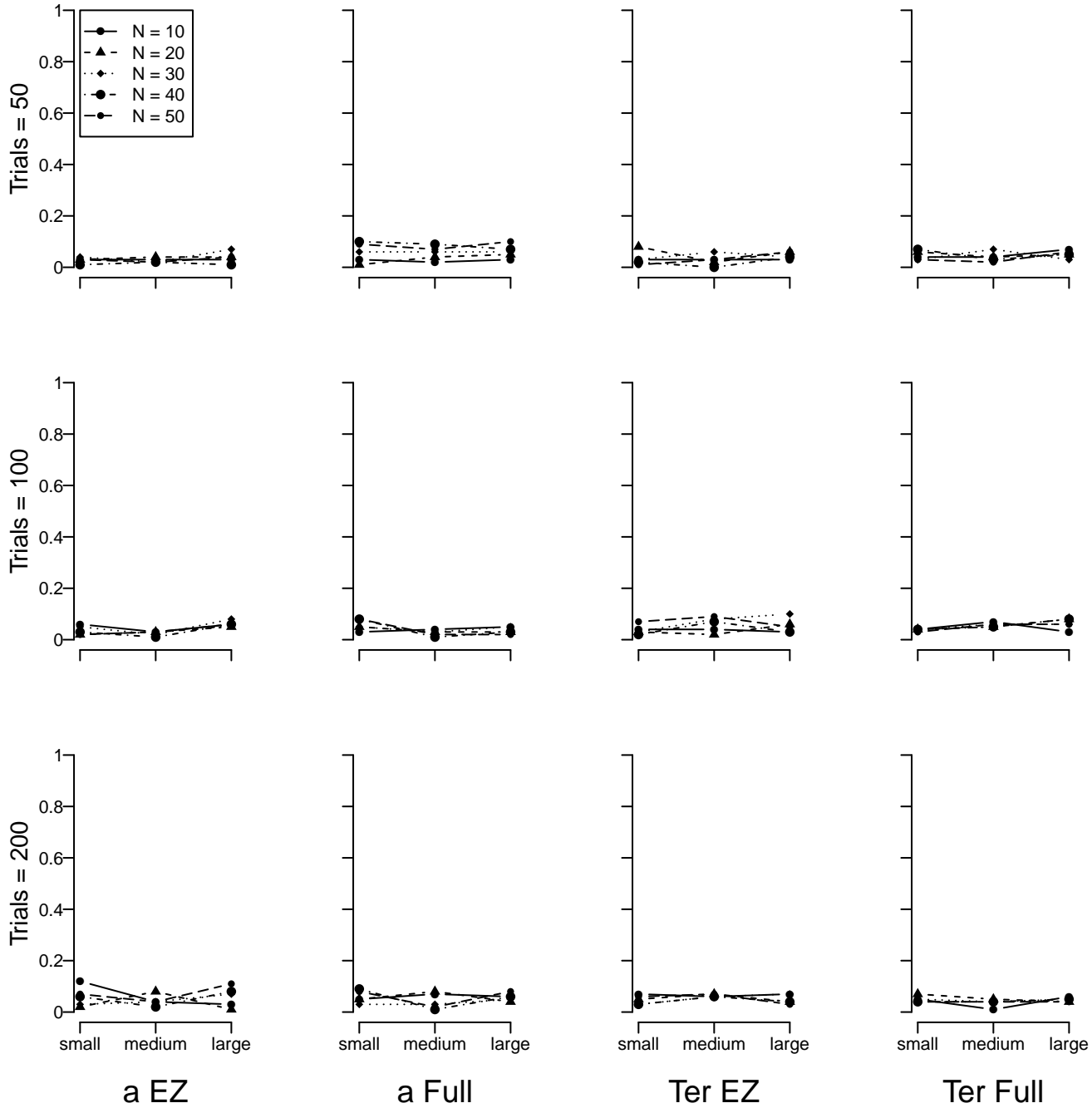

Supplement: Supplementary file 1 — (ZIP 205 KB) [file 13423_2016_1081_MOESM1_ESM.zip › Figures/vFullRes.pdf]

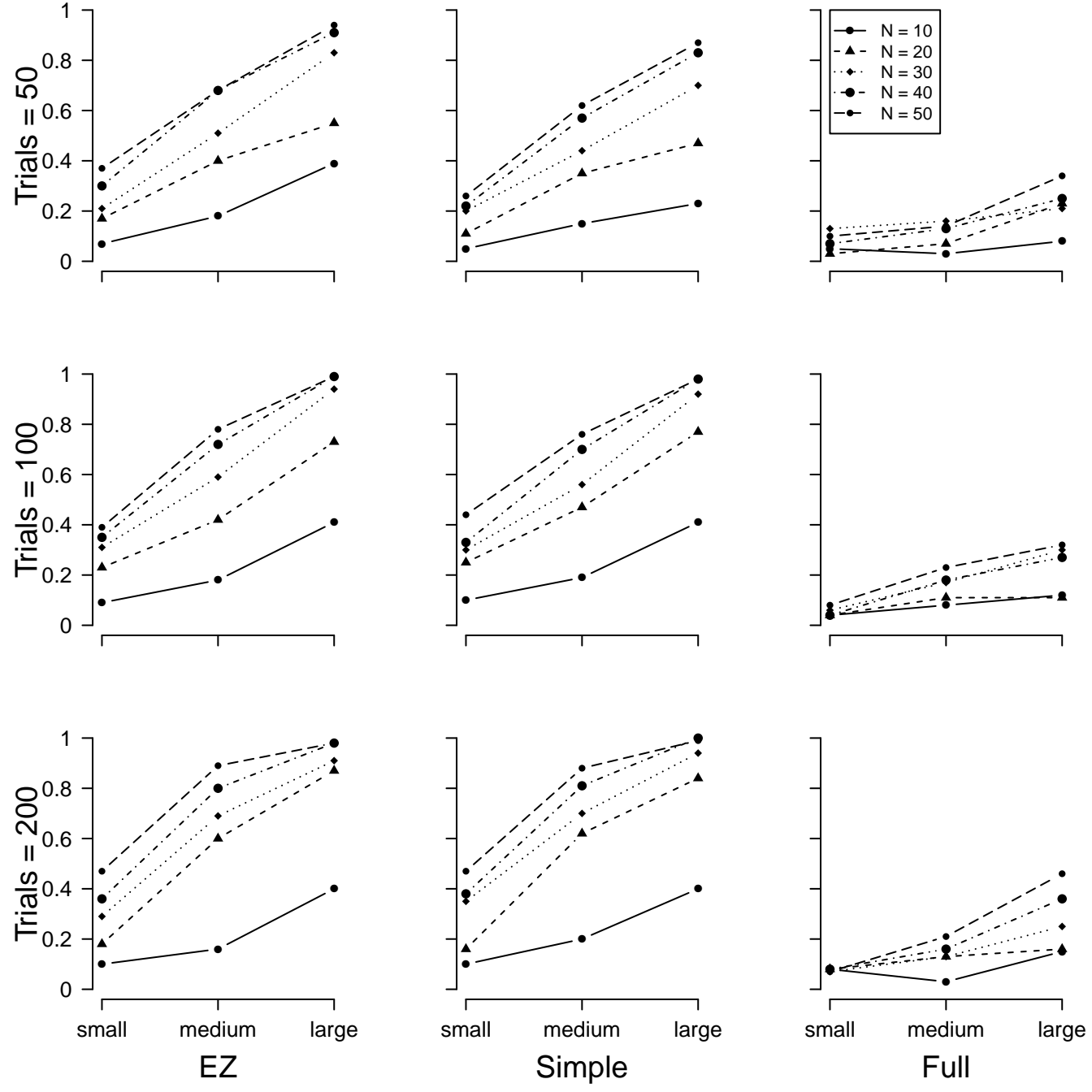

Supplement: Supplementary file 1 — (ZIP 205 KB) [file 13423_2016_1081_MOESM1_ESM.zip › Figures/vSup.pdf]
